# A mathematical model of catalyst combination design and temperature control in the preparation of C_4_ olefins through ethanol coupling

**DOI:** 10.1039/d3ra01363d

**Published:** 2023-04-04

**Authors:** Pengxiang Tang, Hongting Li, Xiaomei Zhang, Xin Sun

**Affiliations:** a School of Pharmacy, Jilin Medical University Jilin Jilin Province 132013 China cxtpxedu@163.com zirenjlcc@jlmu.edu.cn sunxin@jlmu.edu.cn; b Queen Mary College, Nanchang University Nanchang Jiangxi Province 330000 China

## Abstract

The preparation of C_4_ olefins through ethanol catalytic coupling is a crucial area of study. According to the experimental data obtained by a chemical laboratory for different catalysts at different temperatures, three mathematical models were developed to provide insights into the relationships among ethanol conversion rate, C_4_ olefins selectivity, yield, catalyst combination, and temperature. The first model is a nonlinear fitting function that analyses the relationships among ethanol conversion rate, C_4_ olefins selectivity, and temperature under varying catalyst combinations. Two-factor analysis of variance was employed to determine the influence of catalyst combinations and temperatures on ethanol conversion rate and C_4_ olefins selectivity. The second model is a multivariate nonlinear regression model that describes the relationships among the yield of C_4_ olefins, catalyst combination, and temperature. Finally, an optimization model was derived based on the experimental conditions; it provides a solution for the selection of the optimal catalyst combinations and temperatures to achieve the maximum yield of C_4_ olefins. This work has significant implications for the field of chemistry and the production of C_4_ olefins.

## Abstract 2

The preparation of C_4_ olefins through ethanol catalytic coupling is a crucial area of study.

## Graphical abstract

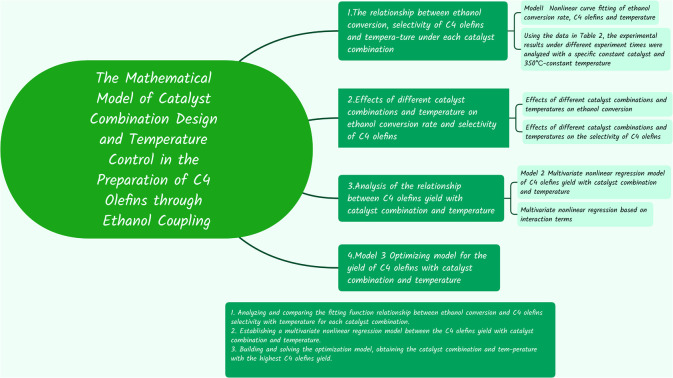

## Introduction

Ethanol is a clean, easily obtained raw material that chemical industry methods and biological fermentation techniques can produce. Biological fermentation is the popular primary technology and uses corn, sugarcane, and other crops as raw materials to produce ethanol.^1^ As the technology matures and the scale of use expands, the industrial application of ethanol as a raw material is also increasing. For example, ethanol is a renewable fuel that can be used in engines.^2^ It can also be used as a coolant in various metal–organic frames and similar applications,^3^ which has broad prospects for producing high-value-added products, such as C_4_ olefins, an essential primary chemical raw material. C_4_ olefins can be obtained through fluid catalytic cracking (FCC) or from byproducts in ethylene cracking reactions. Isobutene can be converted into methyl *tert*-butyl ether (MTBE) by methanol etherification,^4,5^ and is one gasoline additive. The reaction mechanism of preparation of C_4_ olefins by ethanol coupling is the Prince mechanism,^6^ or aldol condensation mechanism.^7^

The preparation of C_4_ olefins through ethanol coupling is very complicated, and the mechanism of the reaction must be further studied. In the preparation of C_4_ olefins through ethanol coupling, it is crucial to control the temperature and catalyst design.^8^ In an experiment in China, Lv^9^ designed a Co/SiO_2_-HAP catalyst with both acid and base activities on the surface that is aimed at the preparation of C_4_ olefins using ethanol. She studied the optimum conditions for the catalyst charging ratio and reaction temperature in a chemical experiment; her conclusions are consistent with those of this article which employs a mathematical modelling method. Ge^10^ studied the selective superposition process of mixed C_4_ olefins using experimental methods and investigated the influence of reaction conditions on the selective superposition of mixed C_4_ olefins, such as temperature, air speed, and pressure. Through such experiments, it has been concluded that the selectivity of C_4_ olefins will be significantly reduced if the temperature drops, which supports the findings obtained from the analysis of experimental data in this paper.

However, using experimental data, the mathematical modelling method can be employed to study the quantitative relationship and optimal design in the preparation of C_4_ olefins through ethanol coupling, which is an interdisciplinary method. Mathematical modelling is widely used in various fields. For example, it has been applied to identify an optimization strategy to improve the performance of microbial fuel cells^11^ and to assess the risk of airborne transmission of COVID-19.^12^ Moreover, it has been used for drug discovery and development.^13^ In examining the preparation of C_4_ olefins by ethanol coupling, Li *et al.*^14^ established the Analytic Hierarchy Process/Entropy Weight Method-Technique for Order Preference by Similarity to Ideal Solution (AHP/EWM-TOPSIS) and built a production-quality C_4_ olefins assessment system. With the support of the evaluation system, the improved mixed congruence method was used to simulate the production conditions of the preparation of C_4_ olefins through ethanol coupling and to construct the reverse neural network (BPNN). Then, the optimal scoring production scheme at different temperatures was determined using the mathematical model. Wang *et al.*^15^ employed a logistic regression model to analyse the relationship between ethanol temperature and conversion rate with C_4_ olefins selectivity in C_4_ olefins preparation through ethanol coupling. The relationship between different catalysts and temperature with the maximum yield of C_4_ olefins was also examined by constructing a neural network. Zhang *et al.*^16^ conducted a two-dimensional visualisation analysis using experimental data on ethanol-coupled C_4_ olefins and used clustering analysis for different catalyst combinations. Finally, a BPNN was used to calculate the reaction conditions for the maximum yield of ethanol-coupled C_4_ olefins. However, these studies have all been conducted from a single point of view, giving us an incomplete and unsystematic understanding of the preparation of C_4_ olefins by ethanol coupling.

Therefore, based on the experimental data collected from the reactions of preparing C_4_ olefins through ethanol coupling, this paper systematically analysed and solved the four-part problem using mathematical modelling. In the first part, based on the characteristics of the experimental data and on the premise of the unknown reaction mechanism, the relationships among the key components, such as ethanol, C_4_ olefins, and temperature, were analysed, and different fitting functions were compared. In the second part, a specific constant catalyst combination and reaction temperature were selected to study the data characteristics of specific indexes of the reaction components under different experimental time, which has further explained how the reaction conditions change over time. In the third part, the influence of varying catalyst combinations and temperatures on the critical indexes of ethanol conversion rate and C_4_ olefins selectivity were analysed using experimental data. In the fourth part, the yield of C_4_ olefins in the reaction was calculated according to the experimental data, and a multivariate nonlinear model of C_4_ olefins yield with catalyst and temperature was established. A reasonable optimisation model was established to find the optimum catalyst combination and corresponding temperature under different charging methods.

The general reaction process of preparing C_4_ olefins through ethanol coupling is as follows:1
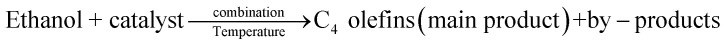


A chemical laboratory has conducted several experiments on the preparation of C_4_ olefins through ethanol coupling. The corresponding experimental data were obtained by changing the experimental conditions of catalyst combination (Co loading, Co/SiO_2_, HAP loading ratio, ethanol concentration) and temperature. In Experimental Data 1, there are 21 groups of catalyst combinations (14 groups of class A, 7 groups of class B). Each group contains five temperatures and the corresponding ethanol conversion rates, ethylene selectivity, C_4_ olefins selectivity, acetaldehyde selectivity, carbon number 4–12 fatty alcohol selectivity, methyl benzaldehyde and methyl benzyl alcohol selectivity, and the experimental data for the selectivity of other products. Experimental Data 2 comprises data of unknown catalyst combinations at 350 °C at six time points and contains the ethanol conversion rate, C_4_ olefins selectivity, and so on. It is of great practical significance to study the influence of changing temporal conditions on C_4_ olefins selectivity and C_4_ olefins yield. It is also important to use existing experimental data and results to analyse and explore the reactions of C_4_ olefins preparation through ethanol coupling.

## Experimental design

### Data sources

The original experimental data used in this paper are from Question B of the 2021 Higher Education Community Cup National Mathematical Contest in Modelling for College Students;^17^ charging method I was used in catalyst experiments A1–A14, and charging method II was used in catalyst experiments B1–B7. Some experimental data are shown in Tables 1 and 2, and the parameters used in this paper are presented in Table 3.

**Table 1:** Experimental Data 1 (catalyst combination, temperature, ethanol conversion rate, experimental selectivity)

Catalyst combination number	Catalyst combination	Temperature (°C)	Ethanol conversion rate (%)	Ethylene selectivity (%)	C_4_ olefins selectivity (%)	Acetaldehyde selectivity (%)
A1	200 mg 1 wt% Co/SiO_2_-200 mg HAP-ethanol concentration 1.68 ml min^−1^	250	2.07	1.17	34.05	2.41
		275	5.85	1.63	37.43	1.42
		300	14.97	3.02	46.94	4.71
		325	19.68	7.97	49.70	14.69
		350	36.80	12.46	47.21	18.66
A2	200 mg 2 wt% Co/SiO_2_-200 mg HAP-ethanol concentration 1.68 ml min^−1^	250	4.60	0.61	18.07	0.94
		275	17.20	0.51	17.28	1.43
		300	38.92	0.85	19.60	2.21
		325	56.38	1.43	30.62	3.79
		350	67.88	2.76	39.10	4.20
A14	33 mg 1 wt% Co/SiO_2_-67 mg HAP-ethanol concentration 1.68 ml min^−1^	250	2.50	0.14	1.89	2.63
		275	5.30	0.14	2.55	2.80
		300	10.2	0.25	3.61	4.07
		350	24.0	1.04	10.83	6.25
		400	53.6	2.92	22.30	7.22
B1	50 mg 1 wt% Co/SiO_2_-50 mg HAP-ethanol concentration 1.68 ml min^−1^	250	1.40	0.10	6.32	5.70
		275	3.40	0.19	8.25	4.03
		300	6.70	0.45	12.28	4.11
		350	19.3	1.22	25.97	4.40
		400	43.6	3.77	41.08	4.13
B7	100 mg 1 wt% Co/SiO_2_-100 mg HAP-ethanol concentration 0.9 ml min^−1^	250	4.40	0.13	4.08	2.04
		275	7.90	0.15	6.62	3.49
		300	11.70	0.20	12.86	6.47
		325	17.80	1.42	18.45	7.94
		350	30.20	1.53	25.05	10.30
		400	69.40	2.51	38.17	13.96

**Table 2:** Experimental Data 2 (given catalyst combinations at 350 °C)

Time (min)	Ethanol conversion rate (%)	Selectivity (%)		
		Ethylene selectivity	C_4_ olefins selectivity	Acetaldehyd*e* selectivity
20	43.50	4.23	39.90	5.17
70	37.80	4.28	38.55	5.60
110	36.60	4.46	36.72	6.37
163	32.70	4.63	39.53	7.82
197	31.70	4.62	38.96	8.19
240	29.90	4.76	40.32	8.42
273	29.90	4.68	39.04	8.79

**Table 3:** Symbols and definitions of the parameters

Parameter	Explanation
*T*	Temperature
*t*	Time
*i*	Combination number
*Ai*	Catalyst combination number using charging method I in Table 1
*Bi*	Catalyst combination number using charging method II in Table 1
*Y*(*T*)	Ethanol conversion rate, corresponding to a specific catalyst combination and temperature (%)
*P*(*T*)	C_4_ olefins selectivity, corresponding to a specific catalyst combination and temperature (%)
*y*_I_	Yield of C_4_ olefins in charging method I
*y*_II_	Yield of C_4_ olefins in charging method II
*x*1	Co load
*x*2	Co/SiO_2_
*x*3	HAP
*x*4	Amount of ethanol added per minute

## The relationships among ethanol conversion rate, selectivity of C_4_ olefins, and temperature under each catalyst combination

The relationships among temperature change and selectivity of ethanol conversion rate and C_4_ olefins are studied in different catalyst combinations. The experimental data in Tables 1 and 2 have been preliminarily analysed using scatterplots. The findings indicate that the temperature changes in different catalyst combinations have some relationships with ethanol conversion rate and C_4_ olefins selectivity. The curve fitting toolbox (cftool) in MATLAB was used for preliminary data fitting.^18^ Through comparing the coefficients of determination, *R*^2^, and the residuals among various fitting functions, a better fitting function was obtained. Then their correlation was analyzed.^19^ Next, the data in Table 2 were classified. Since the time data are not uniformly distributed but are complete, spline interpolation was used to supplement the complete time data. The selectivity data were analysed using scatterplots and were processed according to the data trends.

### Model 1: nonlinear curve fitting of ethanol conversion rate, C_4_ olefins, and temperature

**Fig. 1:**
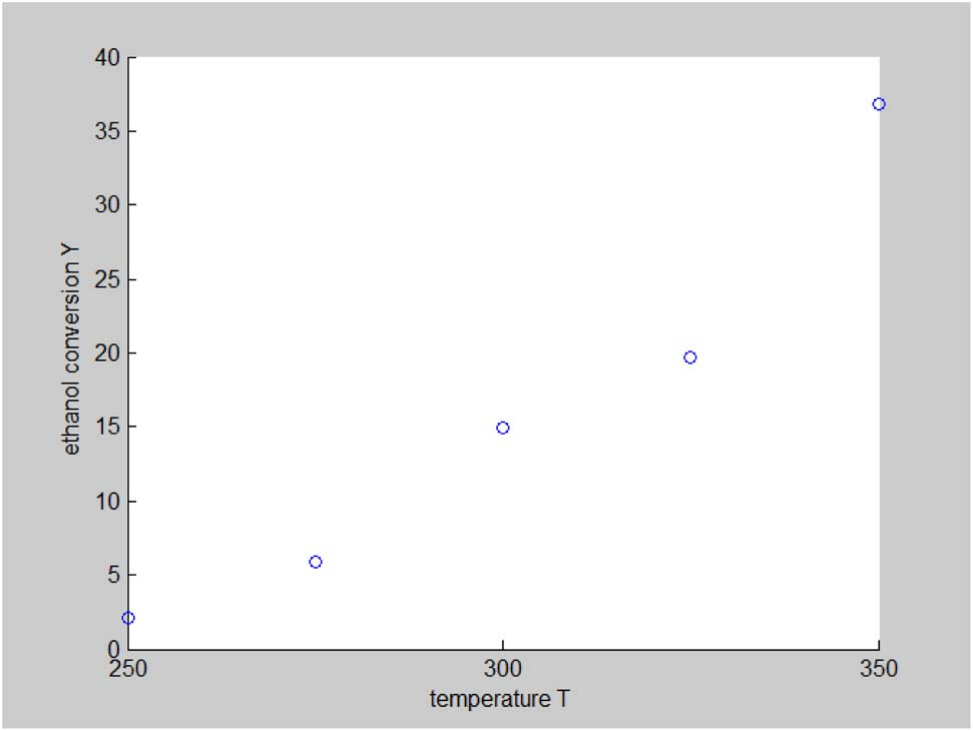
Scatter plot of temperature *T* and ethanol conversion rate *Y* in catalyst group A1.

The original experimental data in Table 1 suggest that the temperature increases from 250 °C in each group of catalysts. There are specific changes in the ethanol conversion rate *Y* and C_4_ olefins selectivity *P*, which were the core elements of the experiment. MATLAB software was used to draw each catalyst combination scatter plot of temperature and ethanol conversion rate. For example, the relationship between temperature *T* and ethanol conversion rate *Y* in catalyst group A1 is shown in Fig. 1.

**Fig. 2:**
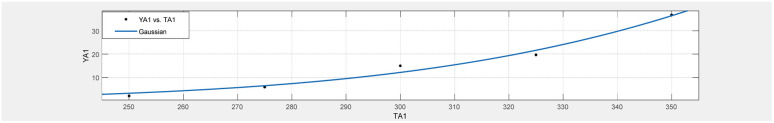
Fitting function diagram of temperature *T* and ethanol conversion rate *Y* in catalyst group A1.

The preliminary analysis of the figure indicates a specific relationship between the temperature *T* of the A1 catalyst and ethanol conversion rate *Y*; the curve fitting toolbox in MATLAB was used for fitting. In the chemical reaction with an unknown mechanism, the most suitable curve model was selected according to the data distribution in the scatter plot.^20^ The known values increased in the change of temperature-to-ethanol conversion rate, which accorded with the exponential model. However, the ethanol conversion rate is unlikely to grow explosively, as in an exponential model, and it is unlikely to exceed or equal 100%, so the exponential model was not adopted. At the beginning the trend of ethanol conversion rate increases with the temperature, and then at a certain point of time, it decreases, and it does not change periodically hence.^21^ Therefore, the relation equation should be obtained by fitting the Gaussian distribution model;^22^ the same is true for the selectivity of C_4_ olefins (Fig. 2).

According to the curve fitting, the relationship between temperature *T* and ethanol conversion rate *Y* satisfied the equation:2
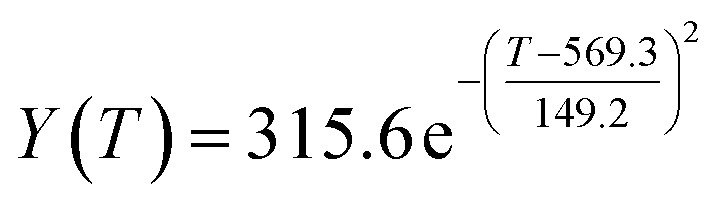


The residual value is 13.6410, and the coefficient of determination, *R*^2^, is 0.9817, indicating an excellent fit.

By comparing the *R*^2^ and residual values, the closer *R*^2^ is to 1, the better, and the smaller the residual value is, the better. Furthermore, considering the simplicity of the equation, the fitting functions of ethanol conversion rate, C_4_ olefins selectivity, and temperature under the other catalyst groups (groups A02–A14 and B01–B07) could be obtained, as shown in Table 4.

**Table 4:** Fitting functions of temperature *T*, ethanol conversion rate *Y*(*T*), and C_4_ olefins selectivity *P*(*T*) in different catalyst combinations

Catalyst combination	Temperature *T* and ethanol conversion rate *Y*(*T*)	Temperature (*T*) and C_4_ olefins selectivity (*P*)
A1	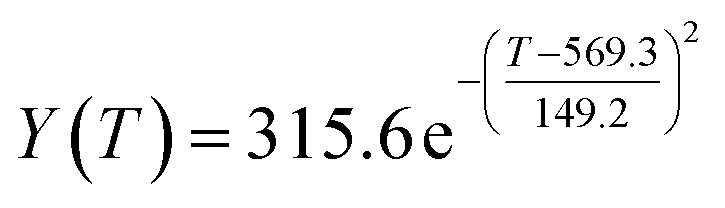	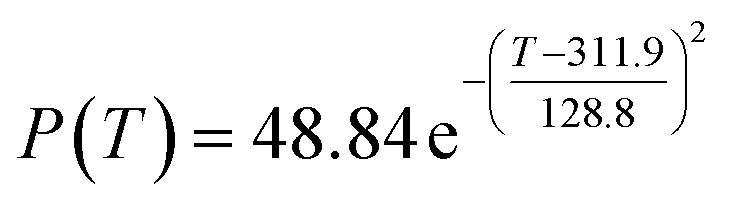
A2	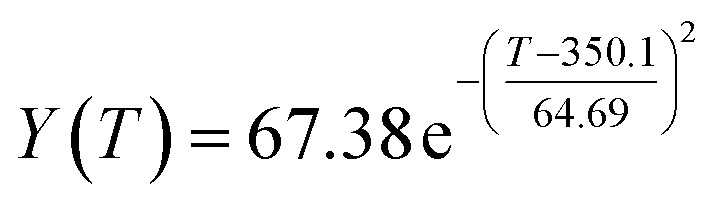	*P*(*T*) = 30.58 − 4.786 cos(*T* × 0.02781) − 13.58 sin(*T* × 0.02781)
A3	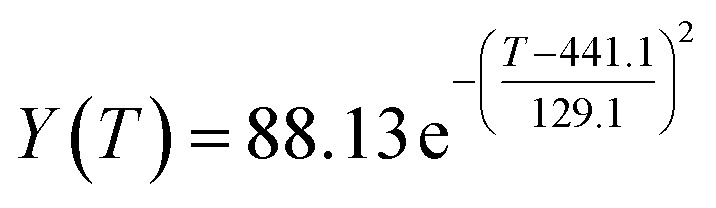	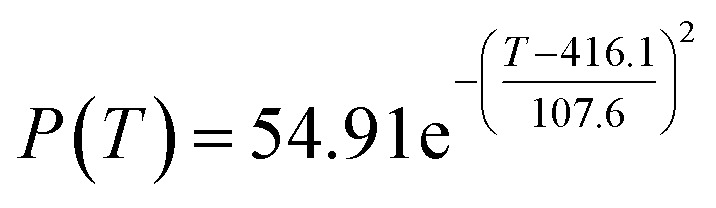
A4	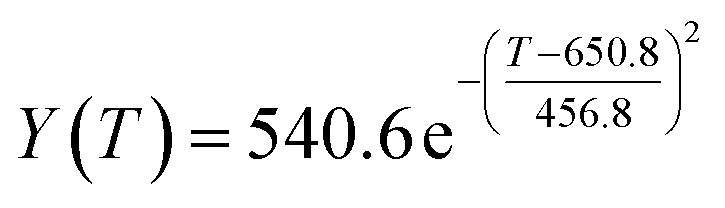	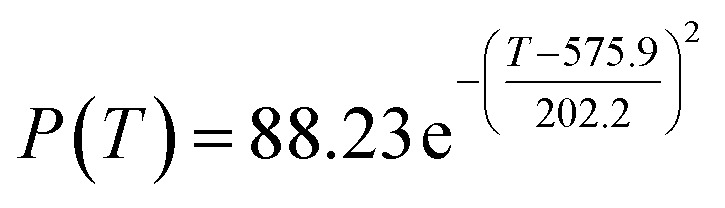
A5	*Y*(*T*) = 436 100 000 − 436 100 000 × cos(*T* × 3.831 × 10^−6^) − 436 500 × sin(*T* × 3.831 × 10^−6^)	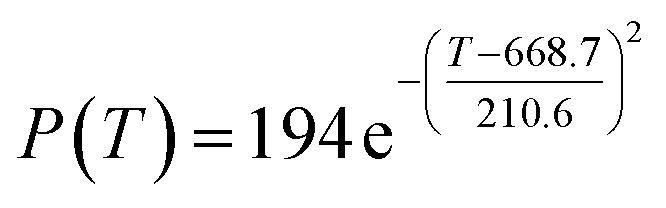
A6	*Y*(*T*) = 0.02233 e^0.01849*T*^	*P*(*T*) = 0.02233 e^0.01849T^
A7	*Y*(*T*) = 29.36 − 74.96 × cos(*T* × 0.005311) + 8.471 × sin(*T* × 0.005311)	*P*(*T*) = 48.03 + 20.86 × cos(*T* × 0.008251) − 36.75 × sin(*T* × 0.008251)
A8	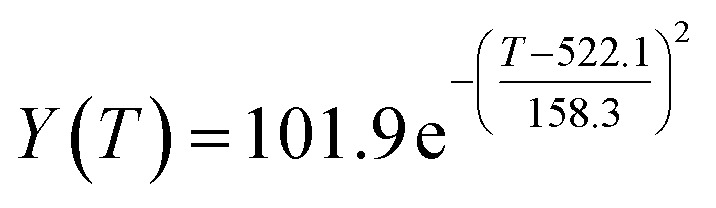	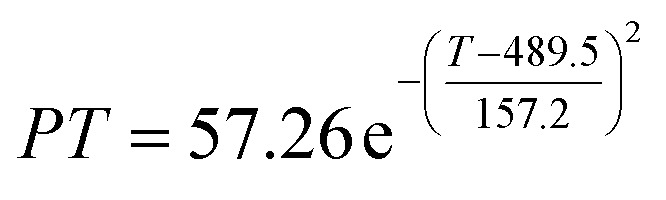
A9	*Y*(*T*) = 0.006922 e^0.0217*T*^	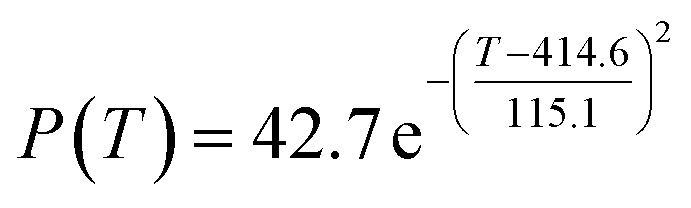
A10	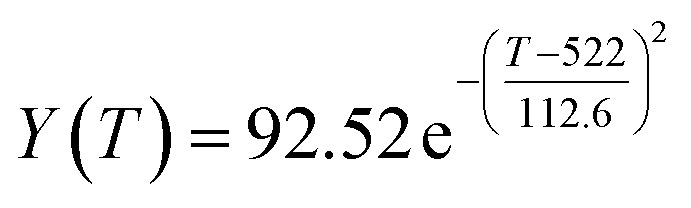	*P*(*T*) = 0.01622 e^0.01605T^
A11	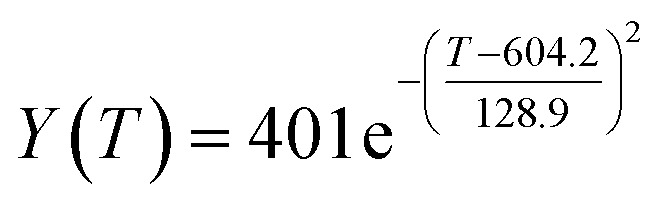	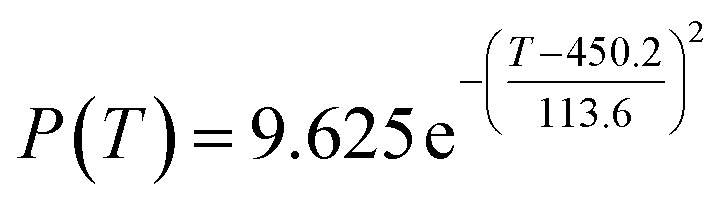
A12	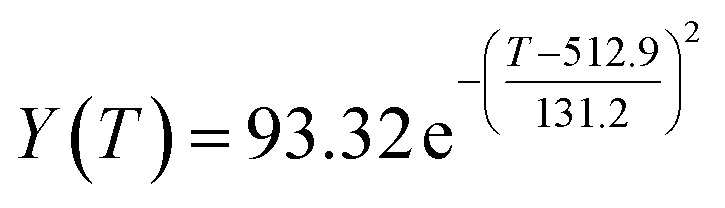	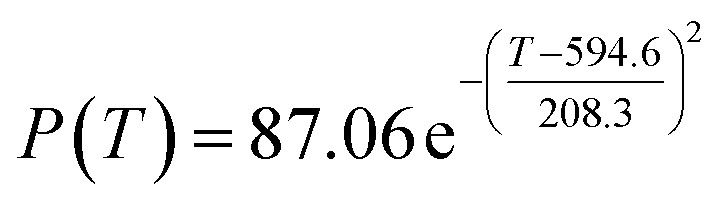
A13	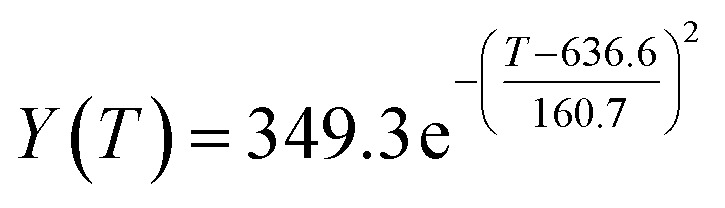	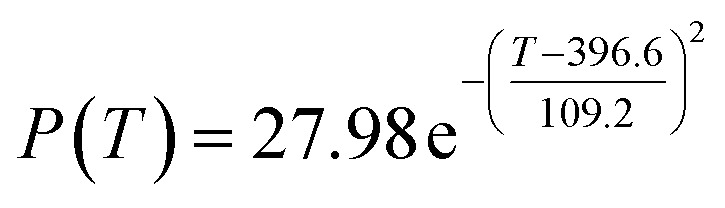
A14	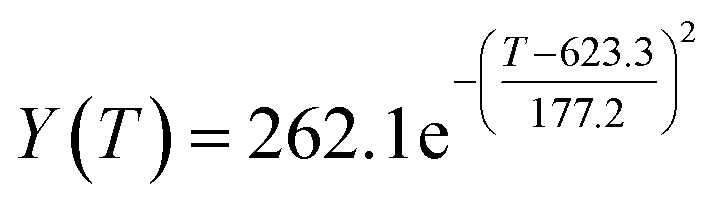	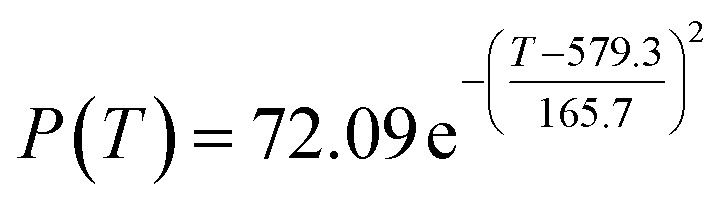
B1	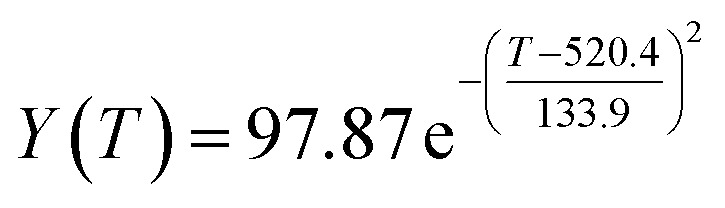	*P*(*T*) = 29.49 + 22.79 × cos(*T* × 0.01355) + 4.235 × sin(*T* × 0.01355)
B2	*Y*(*T*) = 0.01647 e^0.01978*T*^	*P*(*T*) = 0.01647 e^0.01978*T*^
B3	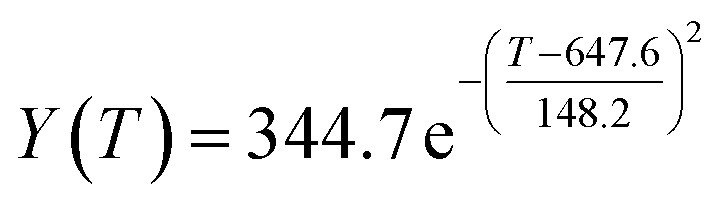	*P*(*T*) = 28.25 + 25.07 × cos(*T* × 0.0129) + 0.401 × sin(*T* × 0.0129)
B4	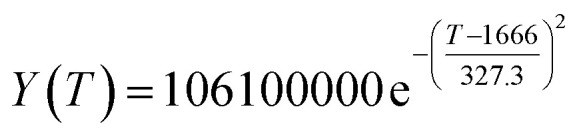	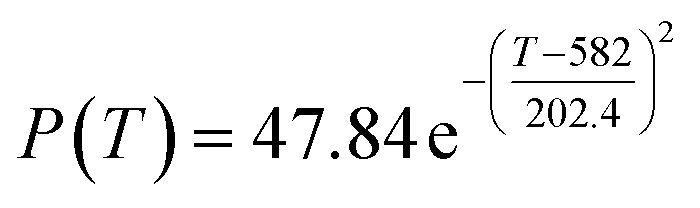
B5	*Y*(*T*) = 0.01256 e^0.02046*T*^	*P*(*T*) = 26.29 + 14.19 ×cos(*T* × 0.009524) − 17.19 × sin(*T* × 0.009524)
B6	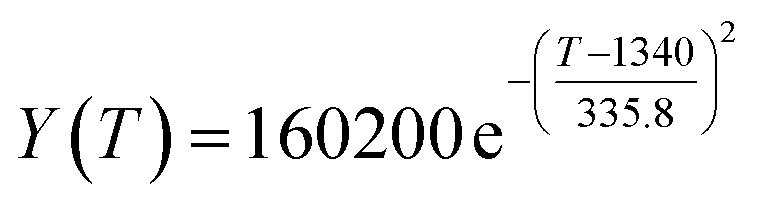	*P*(*T*) = 17.29–11.31 × cos(*T* × 0.02212) + 6.735 × sin(*T* × 0.02212)
B7	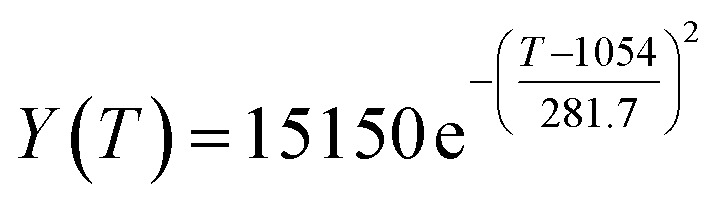	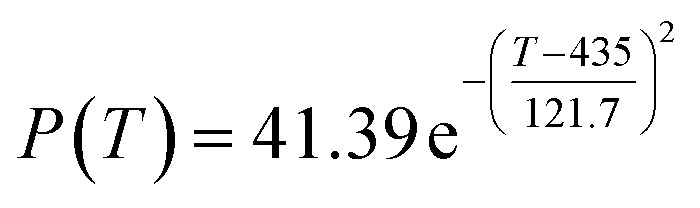

According to the fitting functions in Table 4, the corresponding values of ethanol conversion rate and C_4_ olefins conversion at a given temperature under each catalyst combination can be calculated.

#### Using the data in Table 2, the experimental results under different experiment times were analysed with a specific constant catalyst and 350 °C constant temperature

However, the experiment time in Table 2 is not equally spaced, and the data do not accord with the basic principles of the experiment, so the analysis could not be completed. Therefore, primary treatment should be completed for the data. Using spline interpolation,^23^ starting from 20 minutes, ethanol conversion rates and selectivity indexes were calculated at an isometric time point every 20 minutes. The results are shown in Table 5.

**Table 5:** Experimental Data 2: results of spline interpolation every 20 minutes

Time (min)	Ethanol conversion rate (%)	Selectivity (%)		
		Ethylene selectivity	C_4_ olefins selectivity	Acetaldehyde selectivity
20	43.55	4.23	39.90	5.17
40	39.99	4.21	40.27	5.30
60	38.24	4.25	39.28	5.48
80	37.49	4.32	37.82	5.75
100	36.98	4.41	36.79	6.13
120	35.93	4.51	37.05	6.65
140	34.33	4.59	38.38	7.25
160	32.88	4.63	39.47	8.06
180	32.14	4.62	39.31	8.06
200	31.61	4.63	38.97	8.21
220	30.72	4.69	39.57	8.31
240	29.85	4.76	40.32	8.42
260	29.55	4.76	40.15	8.61
280	30.34	4.61	37.99	8.92

**Fig. 3:**
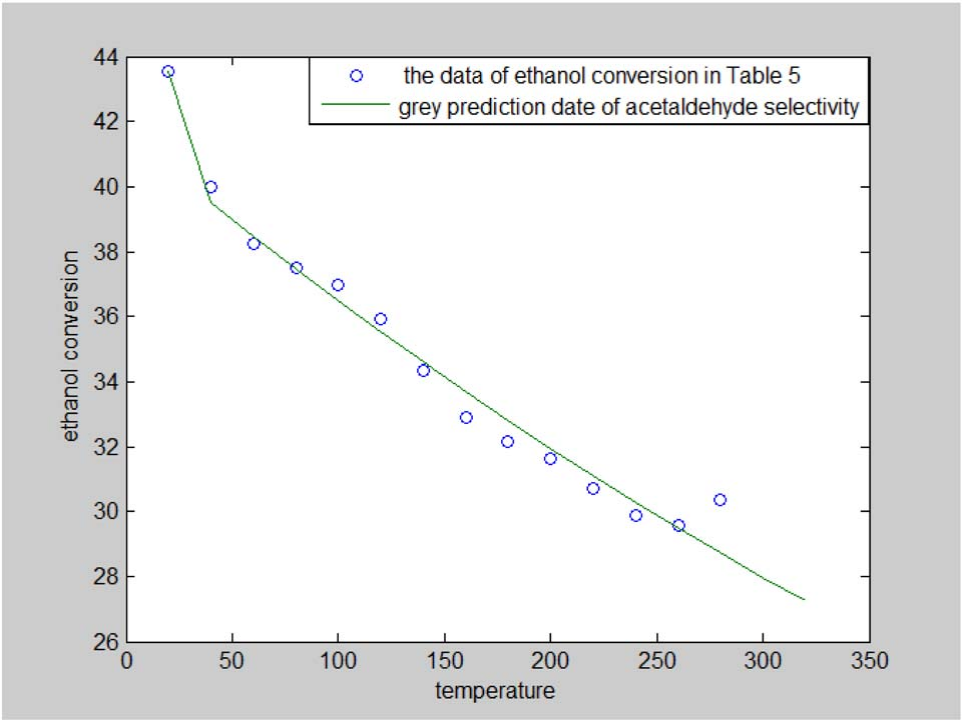
Expectancy map of the grey prediction model for ethanol conversion rate in Table 5.

The results presented in Table 5 suggest that the ethanol conversion rate decreases monotonically with time, and acetaldehyde selectivity increases with time. The other data fluctuate around their means. The grey prediction model GM (1,1)^24,25^ could describe the relationship between time and ethanol conversion rate. It was used to predict the ethanol conversion rate (%); the results are shown in Fig. 3.

Fig. 3 illustrates that the ethanol conversion rate decreased with the increase in reaction time, but the rate of decline also decreased over time. It stabilized at about 29% when the reaction time was 260 minutes.

#### Analysis of ethylene selectivity

**Fig. 4:**
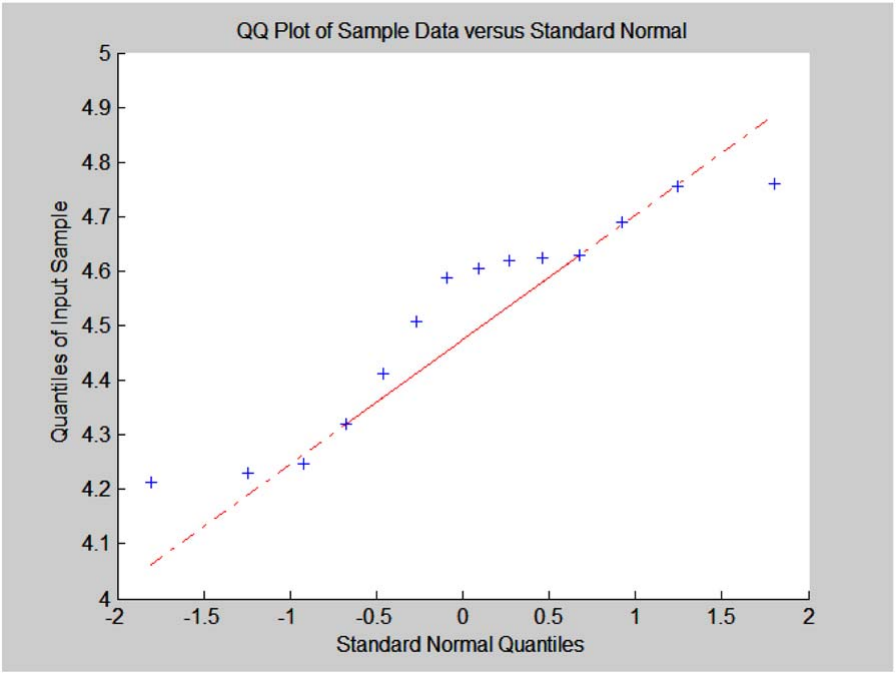
qq diagram of ethylene selectivity in Table 2.

The ethylene selectivity values in Table 5 fluctuate around the mean and are believed to follow a normal distribution, so the qq diagram (Fig. 4) was used for verification.^26^ The distribution of the data points in Fig. 4 is roughly linear, so it can be assumed that the sample data on ethylene selectivity follow a normal distribution, with a mean of 4.51 and a standard deviation of 0.19.

#### Analysis of C_4_ olefins selectivity

**Fig. 5:**
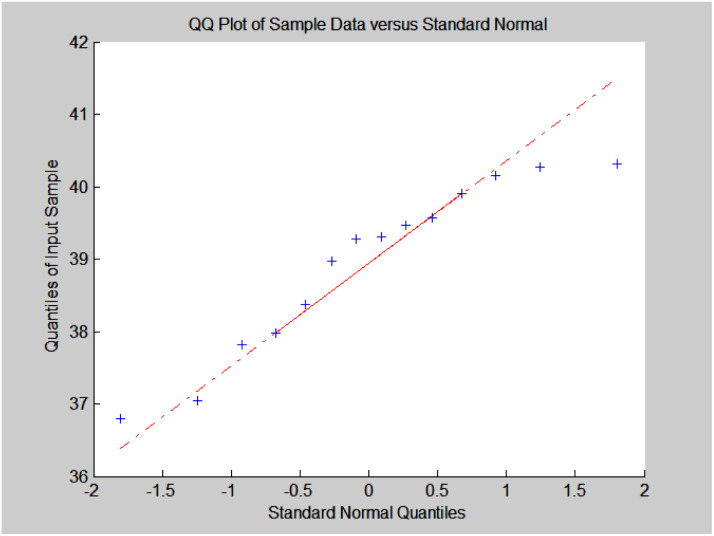
Normal qq verification plot for C_4_ olefins selectivity analysis.

C_4_ olefins selectivity was believed to follow a normal distribution, and the qq plot was used for verification (Fig. 5). The plot appeared linear, so the assumption of normality for the sample data for C_4_ olefins selectivity was supported; the mean is 38.95 and the standard deviation is 1.17.

#### Analysis of acetaldehyde selectivity

**Fig. 6:**
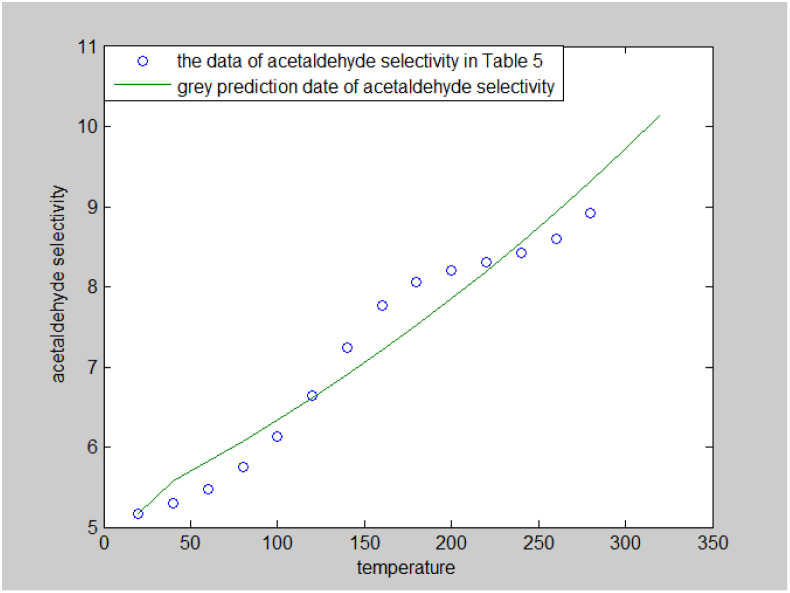
Grey prediction model to predict acetaldehyde selectivity.

The grey prediction model GM (1,1) was used to predict acetaldehyde selectivity (%), and the results are shown in Fig. 6. Here, acetaldehyde selectivity increases with the increase in reaction time, but the rate of change decreases and tends to a stable value of about 9%.

Based on the above analyses, it is clear that ethylene selectivity and C_4_ olefins selectivity are weakly correlated with reaction time.

#### Effects of catalyst combinations and temperature on ethanol conversion rate and selectivity of C_4_ olefins

Table 1 shows that each ethanol conversion rate and C_4_ olefins selectivity are related to different catalyst combinations and temperatures, but the temperature range varies by catalyst. Therefore, the temperature range must be unified before analysis and processing. According to the relationship between temperature and ethanol conversion rate and C_4_ olefins selectivity in different catalyst combinations (obtained using the fitting function in Table 4), the data corresponding to the range 250–400 °C in each group of catalysts were used. For example, using the fitting function in Table 4, the fitting function between ethanol conversion rate and temperature under catalyst combination A1 is as follows:3
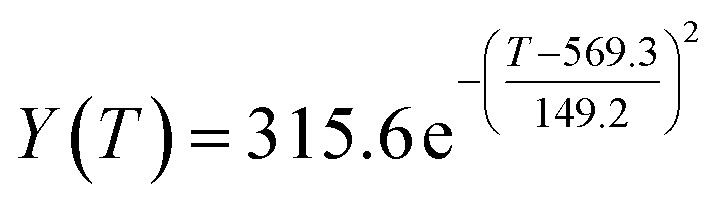


The data for ethanol conversion rate and catalyst combinations at a uniform temperature were obtained.

#### Effects of different catalyst combinations and temperatures on ethanol conversion rate

**Fig. 7:**
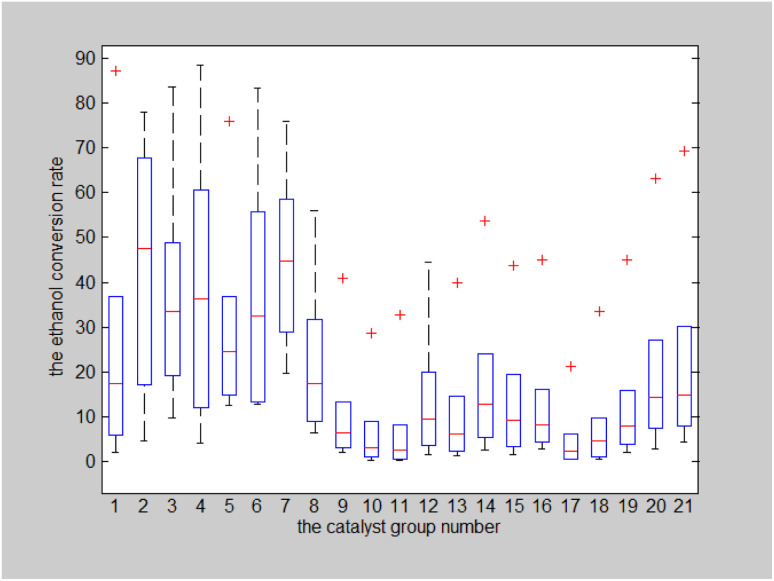
Catalyst combination-temperature-ethanol box diagram. Note: the abscissa represents the catalyst group number, and the ordinate represents the ethanol conversion rate.

The above catalyst combination-temperature-ethanol conversion rate data were imported into MATLAB, and a box diagram was created (Fig. 7).

The mean, maximum, and minimum values of the ethanol conversion rate differ for the 21 catalyst combinations and corresponding temperatures. Moreover, the ethanol conversion rate in charging method I is higher than that in charging method II, which implies that the ethanol conversion rate may be affected by the catalyst combination, temperature, and charging method. To further verify these observations, a two-factor analysis of variance was conducted.^27^ The null hypothesis of no relationship was rejected, as catalyst combination and temperature have a significant effect on ethanol conversion rate (*p* < 0.001).

**Fig. 8:**
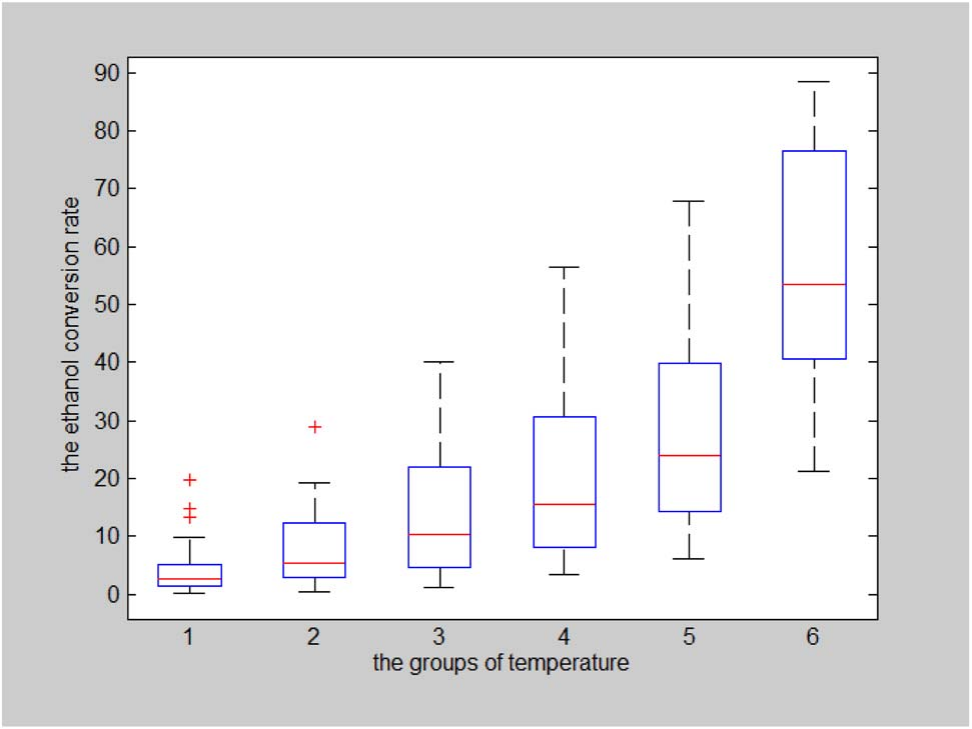
Box plot of ethanol conversion rate for six temperature groups.

Additional analyses were conducted to explore the influence of each temperature group on the ethanol conversion rate using the data in Table 6. A box plot of the ethanol conversion rate for six temperature groups was drawn, as shown in Fig. 8.

**Table 6:** Catalyst combination, temperature, and ethanol conversion rate data

	Ethanol conversion rate at…					
Catalyst combination	250 °C	275 °C	300 °C	325 °C	350 °C	400 °C
A1	2.07	5.85	14.97	19.68	36.80	87.09
A2	4.60	17.20	38.92	56.38	67.88	77.88
A3	9.70	19.20	29.30	37.60	48.90	83.70
A4	4.00	12.10	29.50	43.30	60.50	88.40
A5	14.80	12.40	20.80	28.30	36.80	76.00
A6	13.40	12.80	25.50	39.50	55.80	83.30
A7	19.70	29.00	40.00	49.30	58.60	76.00
A8	6.30	8.80	13.20	21.06	31.70	56.10
A9	2.10	3.00	4.70	8.00	13.40	40.80
A10	0.30	1.00	1.70	4.30	9.00	28.60
A11	0.20	0.50	1.60	3.70	8.20	32.60
A12	1.40	3.50	6.90	12.00	19.90	44.50
A13	1.30	2.30	4.10	8.10	14.60	40.00
A14	2.50	5.30	10.20	15.40	24.00	53.60
B1	1.40	3.40	6.70	11.60	19.30	43.60
B2	2.80	4.40	6.20	10.10	16.20	45.10
B3	0.40	0.60	1.10	3.30	6.00	21.10
B4	0.50	1.10	3.00	6.10	9.60	33.50
B5	2.10	3.80	5.80	9.80	15.90	45.00
B6	2.80	7.50	12.60	15.90	27.00	63.20
B7	4.40	7.90	11.70	17.80	30.20	69.40

**Fig. 9:**
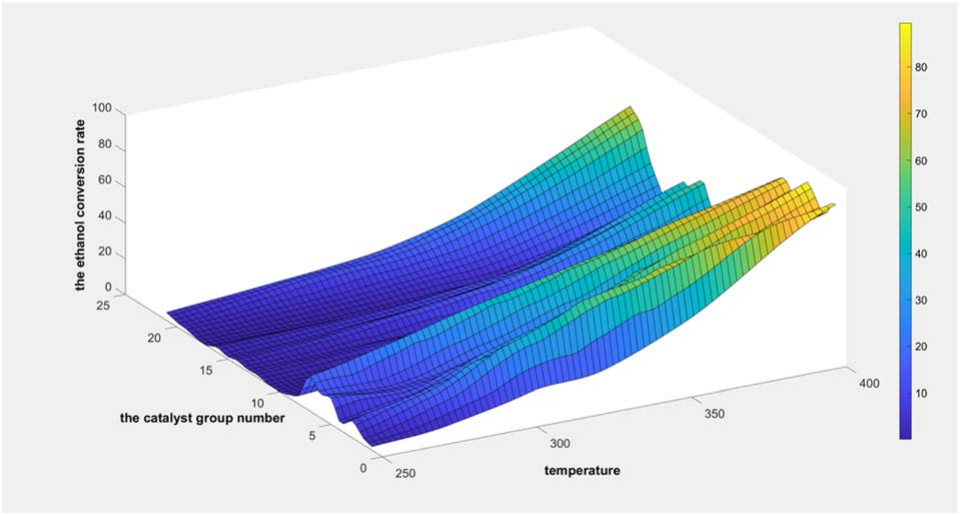
Surface plot of ethanol conversion rate with catalyst combination and temperature.

**Fig. 10:**
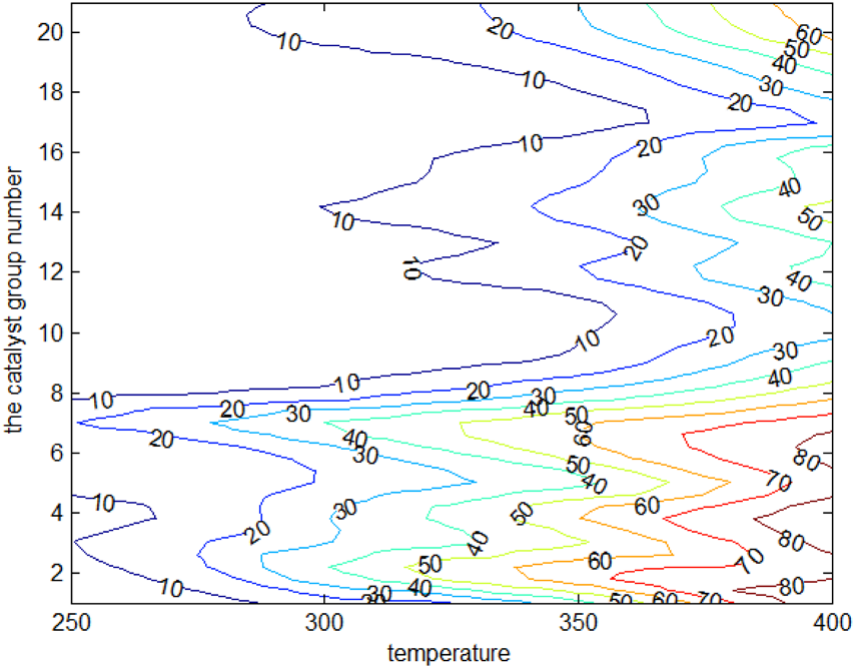
Contour plot of ethanol conversion rate with catalyst combination and temperature.

As indicated in Fig. 8, the ethanol conversion rate is the highest when temperature is high (*t* = 400 °C) and catalyst combination A2 is used. Using two-dimensional interpolation,^28^ the curves for ethanol conversion rate, catalyst combination, and temperature were obtained (Fig. 9 and 10).

Fig. 9 depicts the surface plot of the ethanol conversion rate with catalyst combination and temperature, while Fig. 10 shows the contour plot of the ethanol conversion rate with catalyst combination and temperature. From these illustrations, it is clear that the ethanol conversion rate was highest when the temperature was 400 °C and the catalyst combination was A1, A3, or A6.

#### Effects of catalyst combinations and temperatures on the selectivity of C_4_ olefins

**Fig. 11:**
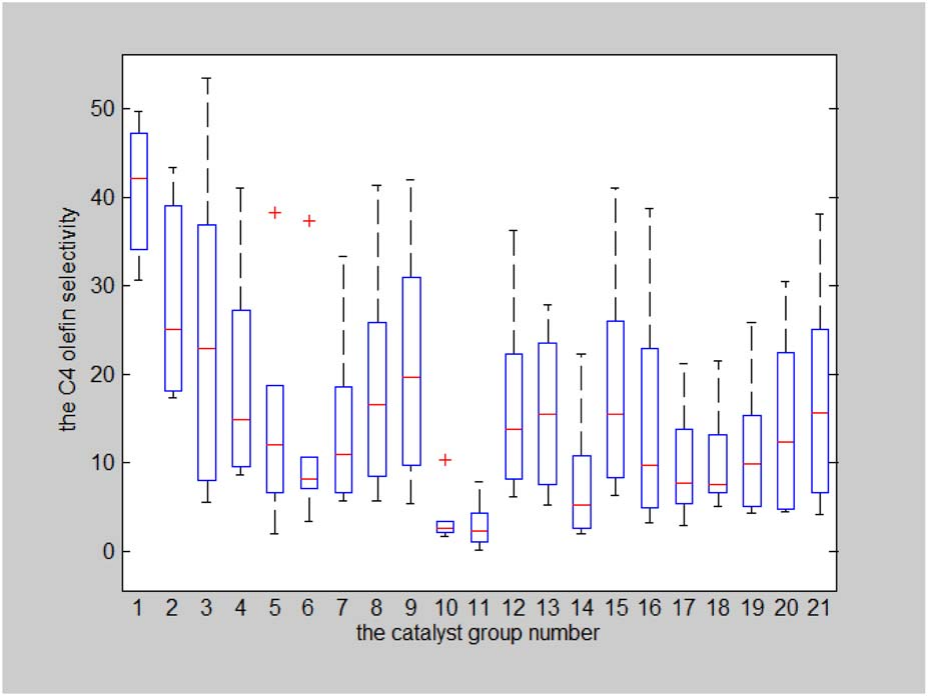
Catalyst combination-temperature-C_4_ olefins selectivity.

The effects of different catalyst combinations and temperatures on the selectivity of C_4_ olefins were analysed using the same approach. The catalyst combination-temperature-C_4_ olefins selectivity box plot is shown in Fig. 11.

The results of a two-factor analysis of variance indicate that the null hypothesis that catalyst combination and temperature have no significant effects on C_4_ olefins selectivity should be rejected (*p* < 0.001 for both).^29^

**Fig. 12:**
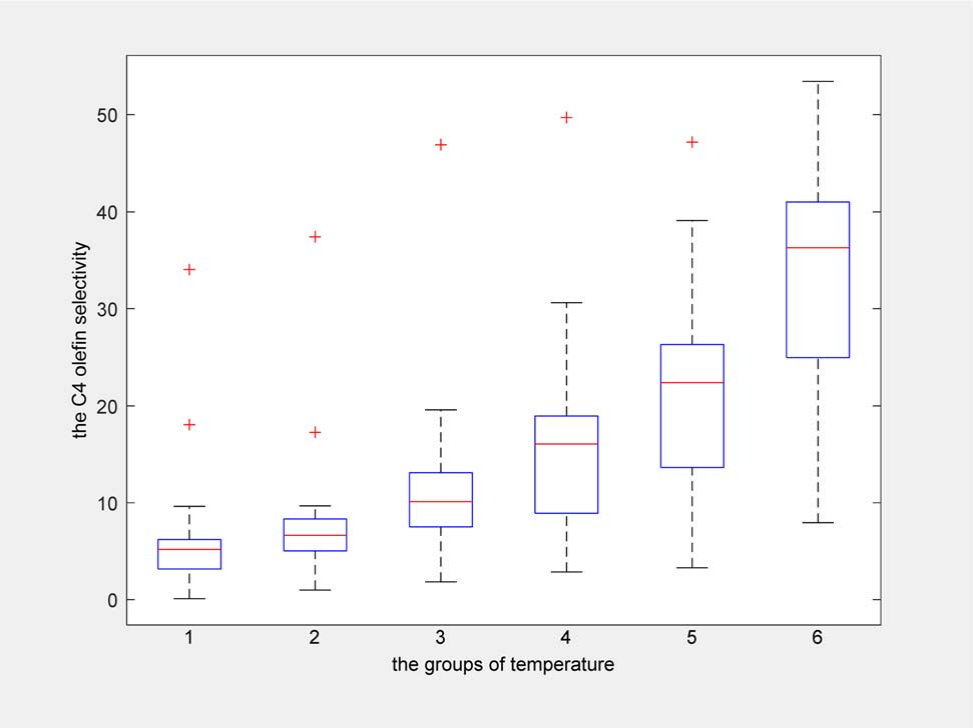
Box plot of C_4_ olefins selectivity for six temperature groups.

As shown in Fig. 12, when the temperature increased, the C_4_ olefins selectivity also increased. When the maximum temperature was 400 °C, the ethanol conversion rate was highest.

**Fig. 13:**
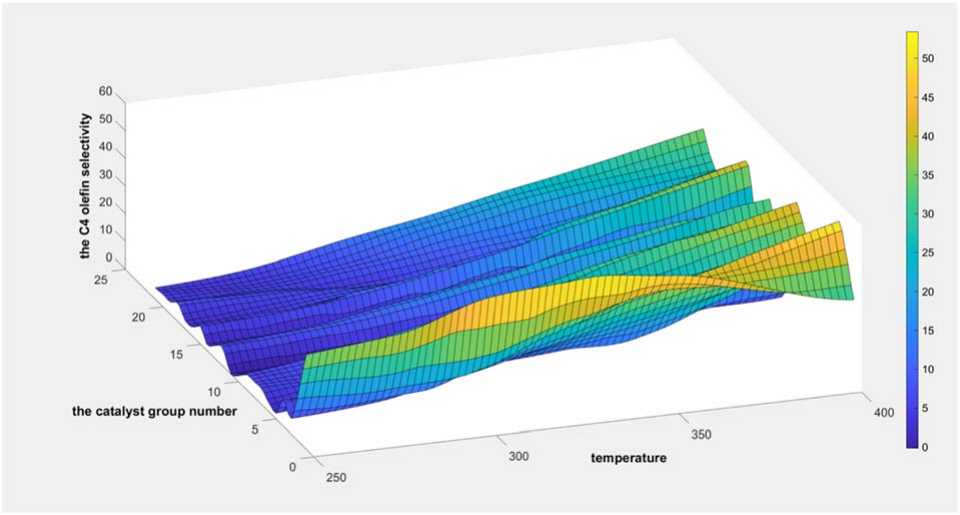
Surface chart of C_4_ olefins selectivity with catalyst combination and temperature.

**Fig. 14:**
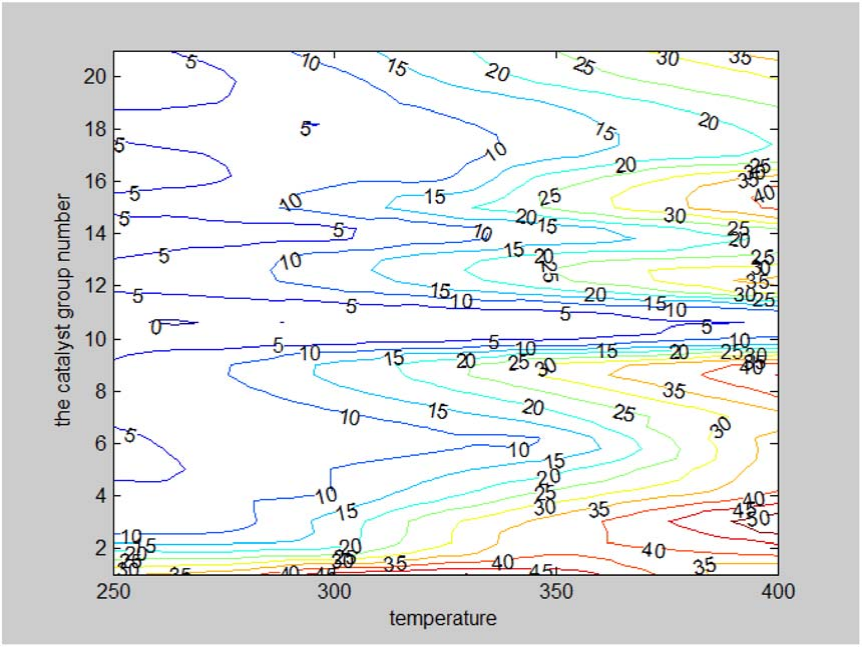
Contour map of C_4_ olefins selectivity with catalyst combination and temperature.

Two-dimensional interpolation was used to create the surface plot (Fig. 13) and contour plot (Fig. 14) for C_4_ olefins selectivity with catalyst combination and temperature. The results indicate that the selectivity of C_4_ olefins is higher when the temperature is 400 °C and the catalyst combination is A2 or A3.

#### Analysis of the relationship between C_4_ olefins yield with catalyst combination and temperature

The yield of C_4_ olefins is the key index in the preparation of C_4_ olefins by ethanol coupling, and the value is equal to the ethanol conversion rate multiplied by the selectivity of C_4_ olefins. The previous analysis showed that catalyst combination and temperature significantly affect the ethanol conversion rate and C_4_ olefins selectivity. Therefore, the catalyst combination and temperature also have a corresponding effect on the C_4_ olefins yield. The quantitative relationship between them was further investigated, and the regression model for C_4_ olefins yield, catalyst combination, and temperature was established.

### Model 2: multivariate nonlinear regression model of C_4_ olefins yield with catalyst combination and temperature

The C_4_ olefins yield equation is as follows:

4 C_4_ olefins yield = ethanol conversion rate × C_4_ olefins selectivity

Using eqn (4) and available data, the yield of C_4_ olefins was calculated, as shown in Table 7.

**Table 7:** Data for catalyst combination-temperature–C_4_ olefins yield

	Yield at…					
Category	250 °C	275 °C	300 °C	325 °C	350 °C	400 °C
A1	0.70	2.19	7.03	9.78	17.37	40.81
A2	0.83	2.97	7.63	17.26	26.54	33.83
A3	0.53	1.55	4.98	10.79	18.03	44.73
A4	0.39	1.04	3.16	8.18	16.48	36.28
A5	0.29	0.83	2.11	3.93	6.90	29.06
A6	0.44	0.91	1.83	3.52	5.94	31.11
A7	1.13	1.91	3.53	6.77	10.92	25.28
A8	0.35	0.75	1.82	4.29	8.21	23.24
A9	0.11	0.29	0.76	1.89	4.16	17.15
A10	0.01	0.02	0.04	0.12	0.30	2.94
A11	0.00	0.01	0.03	0.11	0.36	2.58
A12	0.09	0.28	0.78	2.01	4.43	19.83
A13	0.07	0.18	0.52	1.47	3.43	11.18
A14	0.05	0.14	0.37	1.11	2.60	11.96
B1	0.09	0.28	0.83	2.22	5.01	17.91
B2	0.09	0.22	0.58	1.63	3.70	17.47
B3	0.01	0.03	0.09	0.25	0.47	2.91
B4	0.04	0.07	0.15	0.50	1.26	7.18
B5	0.09	0.19	0.46	1.14	2.43	11.62
B6	0.12	0.36	1.11	2.55	6.06	19.28
B7	0.18	0.52	1.50	3.28	7.57	26.49

The data were normalized, and a multiple linear regression model was established, with the yield of C_4_ olefins as the response variable, and with temperature and four catalysts (Co load, Co/SiO_2_, HAP, ethanol addition per minute) as the predictor variables. The coefficient of determination, *R*^2^, of the multiple linear regression^30^ is only 0.69, which is small, and the optimization results are poor.

#### Multivariate nonlinear regression using interaction terms

According to the results of the multiple linear regression,^31^ it was necessary to analyse the possible nonlinear relationship including an interaction effect between the reaction conditions.^32^

Since the units of temperature, Co loading, Co/SiO_2_, HAP, and ethanol added per minute differ (Table 8), the data for these variables were divided by the corresponding data in the first row in order to remove the units. From previous analyses, multiple interaction effects are known to exist under charging method I, and multiple nonlinear regression was used.^33^ The model can be written as follows:

5 *y*_I_ = 90.384 − 114.04 × *T* − 1.6525 × *x*1 − 136.45 × *x*3 − 9.2553 × *x*4 + 52.928 × *T*^2^ − 3.1473 × *T* × *x*1 + 67.791 × *T* × *x*2 − 27.336*T* × *x*3 − 7.0734*T* × *x*4 − 1.0955 × *x*1^2^ − 102.06 × *x*1 × *x*2 + 104.65 × *x*1 × *x*3 + 8.5108 × *x*1 × *x*4 − 84.468 × *x*2^2^ + 153.35 × *x*2 × *x*3 + 7.0522 × *x*3 × *x*4 + 2.6543 × *x*4^2^

**Table 8:** Experimental data: yield, temperature, and catalyst composition of C_4_ olefins in charging method I

C_4_ olefin yield (y)	Temperature (*x*1)	Co loading capacity (*x*2)	Co/SiO_2_ (*x*3)	HAP (*x*4)	Amount of ethanol added per minute (*x*5)
0.04	250	1	25	25	1.68
0.07	275	1	25	25	1.68
0.15	300	1	25	25	1.68
0.50	325	1	25	25	1.68
1.26	350	1	25	25	1.68
7.18	400	1	25	25	1.68
0.09	250	1	50	50	2.10
0.19	275	1	50	50	2.10
0.46	300	1	50	50	2.10
1.14	325	1	50	50	2.10
2.43	350	1	50	50	2.10
11.62	400	1	50	50	2.10
0.12	250	1	75	75	1.68
0.36	275	1	75	75	1.68
1.11	300	1	75	75	1.68
2.55	325	1	75	75	1.68
6.06	350	1	75	75	1.68
19.28	400	1	75	75	1.68
0.18	250	1	100	100	0.90
0.52	275	1	100	100	0.90
1.50	300	1	100	100	0.90
3.28	325	1	100	100	0.90
7.57	350	1	100	100	0.90
26.49	400	1	100	100	0.90

**Fig. 15:**
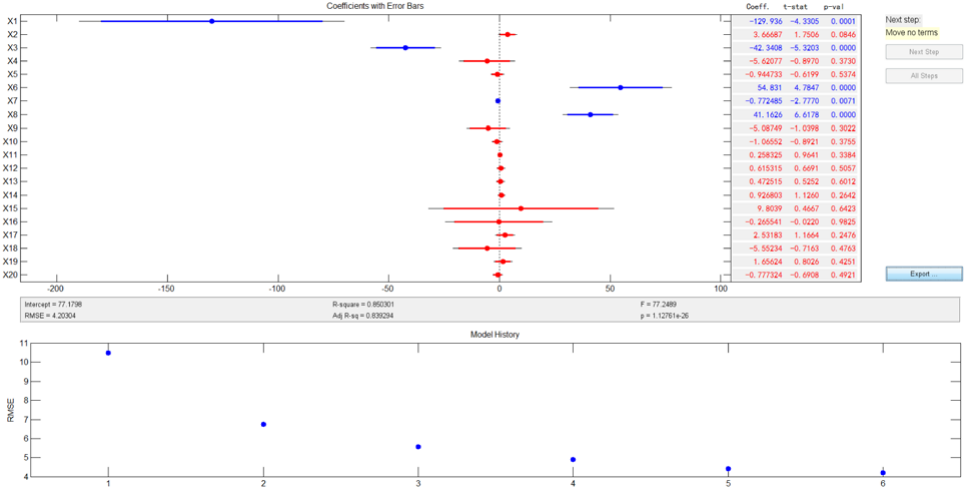
Results of stepwise regression under loading mode I.

The *R*^2^ value is 0.91, indicating that the interaction effects and data nonlinearity in the reaction have a strong fit; however, the model is very complex, which is not conducive to interpreting the results. Therefore, stepwise regression was carried out to further highlight the model's key factors (Fig. 15).^34^ The model for the stepwise regression is:

6 *y*_I_ = 77.1798 − 129.936 × *T* − 2.3408 × *x*2 + 54.831 × *T*^2^ − 0.7725 × *T* × *x*1 + 41.1626 × *T* × *x*2

The results indicate that *R*^2^ is 0.85, further highlighting the key influencing factors and improving the applicability of the model. Moreover, it is concise.

In charging method II, first, based on the results of the multiple linear regression and considering the existence of the interaction effects, group B1 was taken as the benchmark for comparison after removing the units. Complete quadratic polynomial fitting was used to obtain the following model:

7 *y*_II_ = 106.47 − 172.91 × *T* + 69.481 × *T*^2^ + 9.4405 × *T* × *x*2 − 6.445 × *T* × *x*4 − 3.3272 × *x*2^2^ + 0.2338 × *x*2 × *x*4 + 0.80079 × *x*4^2^

The results indicate that the *R*^2^ value is 0.96.

Furthermore, the model using stepwise regression was as follows:

8 *y*_II_ = 115.486 − 181.657 × *T* − 11.557 × *x*2 + 69.48 × *T*^2^ + 10.4993 × *T* × *x*2

**Fig. 16:**
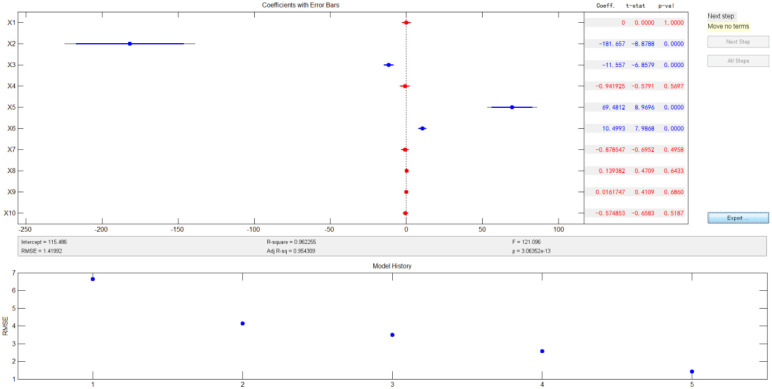
Results of stepwise regression under loading mode II.

The *R*^2^ value is 0.96, and the results are shown in Fig. 16.

### Model 3: optimizing the model for C_4_ olefins yield with catalyst combination and temperature

The optimization model was established using eqn (6) and was constructed as follows:^35^

9 max*y*_I_ = 77.1798 − 129.936 × *T* − 2.3408 × *x*2 + 54.831 × *T*^2^ − 0.7725 × *T* × *x*1 + 41.1626 × *T* × *x*2

The optimized conditions were divided according to the available experimental data:

10 s.t *T* ≥ 1; *T* ≤ 1.8

11 *x*1 ≥ 0.5; *x*1 ≤ 5

12 *x*2 ≥ 0.165; *x*2 ≤ 1

Lingo software was used to identify the optimization solution, and the following results were obtained. Under charging method I, when *T* = 1.6, *x*1 = 0.5, and *x*2 = 1 (*i.e.* when the temperature was 450 °C, the Co load was 0.5 wt%, and the Co/SiO_2_ was 200 mg), the maximum yield of C_4_ olefins was 52%.

When the temperature was below 350 °C, the constraint conditions were changed to identify the optimal solution:^36^

13 *T* ≥ 1; *T* ≤ 1.4

14 *x*1 ≥ 0.5; *x*1 ≤ 5

15 *x*2 ≥ 0.165; *x*2 ≤ 1

Again using Lingo software, the following results were obtained. When the temperature was lower than 350 °C, *T* = 1.4, *x*1 = 0.5, and *x*2 = 1 (*i.e.* when the temperature was 350 °C, the Co load was 0.5 wt%, and the Co/SiO_2_ was 200 mg), the C_4_ olefins yield was at its maximum of 7.48%.

Using eqn (8), an optimization model was built:

16 max*y*_II_ = 115.486 − 181.657 × *T* − 11.557 × *x*2 + 69.48 × *T*^2^ + 10.4993 × *T* × *x*2

17 s.t *T* ≥ 1; *T* ≤ 1.6

18 *x*1 ≥ 1; *x*1 ≤ 4

Lingo software was again used to identify the optimization solution, and the following results were obtained. Under charging method II, when *T* = 1.6 and *x*1 = 4 (*i.e.* temperature was 400 °C and Co/SiO_2_ was 100 mg), the C4 olefins yield reached the maximum of 23.67%. When the temperature was below 350 °C and Co/SiO_2_ was 100 mg, the C_4_ olefins yield reached the maximum of 9.92%.

## Conclusion

In the preparation of C_4_ olefins through ethanol catalytic coupling, the ethanol conversion rate and C_4_ olefins selectivity are two core indexes. The results of this study indicate that the fitting function between ethanol conversion rate, C_4_ olefins selectivity, and temperature under each catalyst combination predicted the values of ethanol conversion rate and C_4_ olefins selectivity under different temperatures. The two-factor analysis of variance showed that different catalyst combinations and temperatures had significant effects on ethanol conversion rate and selectivity of C_4_ olefins. However, analysing the test results under a given catalyst combination at 350 °C at different times in an experiment indicated that the ethylene selectivity and C_4_ olefins selectivity correlate less as reaction time increases. Therefore, the catalyst combination and reaction temperature are mainly considered when analysing the above indexes. To find a certain catalyst combination and temperature that will achieve the highest C_4_ olefins yield under the same experimental conditions, a multiple nonlinear regression model and stepwise regression model of C_4_ olefins yield with four catalysts and temperatures were established and the goal function in the optimization model was obtained. Then, constraint conditions were given under laboratory conditions. Finally, the maximum C_4_ olefins yield was obtained.

Through the establishment and analysis of three mathematical models, this research showed that both catalyst combination and reaction temperature would affect the C_4_ olefins yield. Moreover, the higher the reaction temperature, the higher the yield of C_4_ olefins. The influence of Co loading and Co/SiO_2_ on the yield of C_4_ olefins is greater than that of the other two catalysts. When the minimum of Co loading was 0.5 wt% and the maximum of Co/SiO_2_ was 200 mg, the yield of C_4_ olefins was largest. The amount of ethanol added per minute had little effect on the C_4_ olefins yield.

Based on the experimental data, this paper established a mathematical model and concluded that the higher the reaction temperature, the higher the C_4_ olefins yield. However, when the reaction temperature is higher than the maximum value of 400 °C in the experimental data, will the C_4_ olefins yield continue to increase? And when the temperature continually rises, will the four catalysts undergo denaturation? There are insufficient experimental data to answer these questions, both of which need further study.

## Data availability

The data in this paper come from Question B of the 2021 Chinese Contemporary Undergraduate Mathematical Contest in Modelling (CUMCM).

## Author contributions

Conceptualization, methodology, software, validation, formal analysis, data curation, writing—original draft preparation, visualization, P. T.; writing—review and editing, H. L.; supervision, project administration, funding acquisition, X. Z. and X. S. All authors have read and agreed to the published version of the manuscript.

## Conflicts of interest

There are no conflicts to declare.

## Supplementary Material

## References

[cit1] Wu B., Wang Y. W., Dai Y. H., Song C., Zhu Q. L., Qin H., Tan F. R., Chen H. C., Dai L. C., Hu G. Q., He M. X. (2021). Renewable Sustainable Energy Rev..

[cit2] Mendiburu A. Z., Lauermann C. H., Hayashi T. C., Marinos D. J., da Costa R. B. R., Coronado C. J. R., Roberts J. J., de Carvalho J. A. (2022). Energy.

[cit3] Rupam T. H., Rocky K. A., Palash M. L., Saha B. B. (2023). Therm. Sci. Eng. Prog..

[cit4] Ershov M. A., Potanin D. A., Tarazanov S. V., Abdellatief T. M. M., Kapustin V. M. (2020). Energy Fuels.

[cit5] Nicolaides C. P., Stotijn C. J., Vanderveen E. R. A., Visser M. S. (1993). Appl. Catal., A.

[cit6] Madeira F. F., Gnep N. S., Magnoux P., Maury S., Cadran N. (2009). Appl. Catal., A.

[cit7] Jones M. D., Keir C. G., Di Iulio C., Robertson R. A. M., Williams C. V., Apperley D. C. (2011). Catal. Sci. Technol..

[cit8] Yuan F., Zhang G. H., Zhu J., Ding F. S., Zhang A. F., Song C. S., Guo X. W. (2021). Catal. Today.

[cit9] Lu S. , Master's degree, Dalian University of Technology, 2018

[cit10] Ge Y. , Master's degree, East China University of Science and Technology, 2019

[cit11] Jadhav D. A., Carmona-Martinez A. A., Chendake A. D., Pandit S., Pant D. (2021). Bioresour. Technol..

[cit12] Mittal R., Meneveau C., Wu W. (2020). Phys. Fluids.

[cit13] Sun M. Y., Zhao S. D., Gilvary C., Elemento O., Zhou J. Y., Wang F. (2020). Briefings Bioinf..

[cit14] Li M. H., Zhao L. L., Jin S., Li D. L., Liu J. X. (2022). Heliyon.

[cit15] Wang S., Jiang Z. H., Yang J., Tang Y. L., Liu B. (2022). Energy Rep..

[cit16] Zhang S.-M., Zhan W.-L., Hu H., Liu Y.-S., Zhu J.-M. (2022). J. Chem..

[cit17] C. S. f. I. A. Mathematics, Question B of the 2021 Chinese Contemporary Undergraduate Mathematical Contest in Modeling, http://www.mcm.edu.cn/html_cn/node/4d73a36cc88b35bd4883c276afe39d89.html

[cit18] Sun C. L., Liu M. Z., Ge S. H. (2022). Appl. Sci..

[cit19] Jiang X. G., Wei S. S., Ji J. B., Liu F. F., Li P., Liu C. Y. (2018). Artery Res..

[cit20] Yu L. Y., Wang X. Y., Hou Z. Y., Du Z. Y., Zeng Y. F., Mu Z. Y. (2021). Appl. Sci..

[cit21] Chen Y. W., Hsu Y. H. (2021). Catalysts.

[cit22] Zhang L. L., Oh S. K., Pedrycz W., Yang B., Han Y. M. (2021). Appl. Soft. Comput..

[cit23] Rodinkova V., Mokin V., Vuzh T., Dratovanyj M. (2021). Aerobiologia.

[cit24] Salhein K., Ashraf J., Zohdy M. (2021). Energies.

[cit25] Wang H., Wang Y. H., Wu D. D. (2022). Grey Syst..

[cit26] Sohn W., Hong E. H. (2021). Nucl. Eng. Technol..

[cit27] Cloutman D. G., Jackson D. C. (2003). Fish. Manag. Ecol..

[cit28] Anton F., Mioc D., Fournier A. (2001). Visual Comput..

[cit29] Wu S. S., Hu X. L., Zheng W. B., He C. C., Zhang G. C., Zhang H., Wang X. (2021). Bull. Eng. Geol. Environ..

[cit30] Xie X. F., Wu T., Zhu M., Jiang G. J., Xu Y., Wang X. H., Pu L. J. (2021). Ecol. Indic..

[cit31] Oliveira S., Oehler F., San-Miguel-Ayanz J., Camia A., Pereira J. M. C. (2012). For. Ecol. Manag..

[cit32] Shah V., Jagupilla S. C. K., Vaccari D. A., Gebler D. (2021). Water.

[cit33] Goh A. T. C., Zhang W. G. (2014). Eng. Geol..

[cit34] Radovanovic S., Milivojevic M., Stojanovic B., Obradovic S., Divac D., Milivojevic N. (2022). Appl. Sci..

[cit35] Enikeeva L. V., Faskhutdinov A. G., Koledina K. F., Faskhutdinova R. I., Gubaydullin I. M. (2021). React. Kinet. Mech. Catal..

[cit36] Koledina K. F., Gubaydullin I. M., Koledin S. N. (2022). React. Kinet. Mech. Catal..

